# Quality of life and distress assessed with self and external assessment screening tools in patients with hematologic malignancies attending treatment in an acute hospital

**DOI:** 10.1007/s11136-020-02602-6

**Published:** 2020-08-19

**Authors:** Bianca Senf, Kirsten Grabowski, Natascha Spielmann, Jens Fettel

**Affiliations:** Department of Psycho-Oncology, University Cancer Center (UCT), Johann Wolfgang Goethe University, Theodor-Stern-Kai 7, 60590 Frankfurt/Main, Germany

**Keywords:** Acute hospital, Distress screening, External-/self-assessment, Hematologic malignancies, Psycho-oncology, Quality of life

## Abstract

**Purpose:**

In this study, we examined distress levels and quality of life (QoL) of patients with hematologic malignancies under treatment in an acute setting. We used external- and self-assessment instruments for distress. Additionally, we investigated the relation between distress and QoL as well as whether highly distressed patients differed from less distressed patients concerning their QoL.

**Methods:**

A cross-sectional study with patients of the Medical Clinic II of the University Hospital Frankfurt was conducted. One hundred and nine patients were assessed with an expert rating scale and completed self-report questionnaires. Data were exploratively analyzed and group comparisons between patients who scored above the cut-off of the respective screening instruments and those who did not were conducted.

**Results:**

Patients with hematologic malignancies experience high levels of distress and low QoL. Especially, role and social functioning are affected. Patients suffer most from fatigue, appetite loss, and insomnia. Using established cut-offs, all screening instruments were able to differentiate between patients regarding distress and QoL. Patients scoring above the cut-off were significantly more distressed and had a lower QoL. There was a medium-to-strong correlation between distress and QoL indicators.

**Conclusion:**

Cancer-specific screening instruments seem to be able to identify treatment needs more specifically. They also allowed a better differentiation concerning QoL. The close link between distress and QoL needs to be recognized to enable a holistic approach to treatment and thereby optimize the quality of treatment.

## Introduction

The proportion of hematologic malignancies in all malignant diseases in Germany is currently 7.6% [1]. In comparison to solid tumors, this might seem relatively low, nevertheless, the psychic and social burden inflicted by those diseases is often enormous [2]. Hematologic malignancies include Hodgkin's disease, non-Hodgkin lymphomas, leukemia, and multiple myeloma. The malignant diseases of the lymphatic and hematopoietic system present as a heterogeneous group with regard to their epidemiology and prognosis [3]. On the other hand, there are significant similarities especially concerning distress that, from a psycho-oncological point of view, justify a comprehensive consideration. Distress in cancer patients is defined as “a multifactorial unpleasant emotional experience of a psychological (cognitive, behavioral, emotional), social, and/or spiritual nature that may interfere with the ability to cope effectively with cancer, its physical symptoms, and its treatment.” Distress can range from normal feelings of vulnerability, sadness, and fears to disabling problems (depression, anxiety, etc.) [4]. In some studies, distress is operationalized simply as symptoms of anxiety and depression [5–7]. Chemotherapy is the primary treatment modality for these diseases, and is often associated with serious side effects. Manitta et al. were able to show that the symptom burden and distress of patients with hematologic malignancies correspond to that reported in metastasized patients with non-hematological cancer [2]. Linden et al. investigated the prevalence of anxiety and depression in different tumor entities. Patients with hematologic malignancies had above average distress levels and were among those patients most severely affected by anxiety and depression [6]. Almost 50% of these patients were sub-clinically burdened by anxiety and 23% showed clinically relevant symptoms. The prevalence of depressive symptoms was slightly less with 38% and 17%, respectively. Comparably high prevalence rates are also reported in other studies [8–11]. This is of particular importance as elevated distress levels are associated with an increased risk of mortality in addition to numerous other deleterious effects [7, 12–14]. Batty et al. showed that leukemia patients with elevated distress scores had a higher mortality rate than those with lower distress scores (HR 3.86, 95% CI 1.4–10.5) [7]. The effect persisted even after the authors controlled for a range of sociodemographic variables. Studies show a close association between the extent of distress and quality of life (QoL) in patients with hematologic malignancies [15–18]. To date, there are only a few studies that specifically address the relationship between distress and QoL in patients with hematologic malignancies during acute treatment [16, 17]. It was, therefore, the aim of this study to assess distress with the help of different screening instruments and investigate the relationship between distress and QoL in patients with hematologic malignancies during their treatment in the hospital. The explicit focus on screening instruments reflects the modalities of the daily routine in an acute hospital, where the inpatient treatment of this patient population usually takes place and where screening for distress has to be efficient and uncomplicated [19]. Screening instruments are to be used to efficiently determine the presence of clinically relevant symptoms in a population, in order to reach a decision whether an indication for further psycho-oncological treatment exists. We, therefore, compared how many patients were classified as highly distressed (i.e., scoring above the respective cut-offs) by the three different screening instruments (PO-Bado-SF, HADS-D, and QSC-R10) and, therefore, had an indication for further psycho-oncological treatment. Psycho-oncological treatment in this respect means further diagnostics, psycho education, and if necessary counseling and/or further referral to psychosocial and psychotherapeutic service providers [19]. Lastly, we looked at whether patients that scored above the cut-off values of at least one instrument (highly distressed patients) differed from less distressed patients (patients scoring below the cut-off in all screening instruments) regarding QoL.

## Methods

### Participants

The participants were patients of the Medical Clinic II of the University Hospital Frankfurt who were in treatment for hematological malignancies during the period from April 2017 to November 2018. Included were all patients who fulfilled the following criteria: agreement to participate in the study after obtaining informed consent, age ≥ 18 years, sufficient knowledge of German, no current psychiatric disorder, no medication influencing mental or cognitive abilities, and no acute pain or nausea.

### Procedures

Patients were visited by the staff of the psycho-oncology department and informed in detail about the aims and procedure of the study as well as data collection and possible risks and side effects. After sufficient time for consideration (at least 24 h), the written consent to participate in the study was obtained. Afterwards, trained staff members collected demographic data, carried out the psycho-oncological basic documentation short form (PO-Bado-SF), and recorded the desire for psychosocial support. The remaining instruments (HADS-D, QSC-R10, EORTC QLQ-C30) were then handed over to the patients and an appointment for their return was arranged. The study protocol had previously been approved by the Ethics Committee of the University Hospital Frankfurt (file no. 36/17; decision no. E 25/17).

### Study measures

#### PO-Bado-SF

The psycho-oncological basic documentation short form is a screening instrument that assesses the subjectively perceived distress of cancer patients within the last three days as an external assessment. It is administered in the form of a semi-structured interview, which takes about 5–10 min. Distress is measured with six items on two dimensions: physical and mental. The assessment is based on a five-stage Likert scale from 0 = not stressful to 4 = very stressful [20, 21]. A decision for further psycho-oncological treatment can be reached on the basis of a cut-off value (sum score ≥ 8) [22].

#### HADS-D

The Hospital Anxiety and Depression Scale – German Version (HADS-D) is a 14 items screening measure that assesses self-reported anxiety and depression in somatically ill patients. The time required for completion is approximately 2–6 min. Seven items each form the two subscales for anxiety and depression. Patients are asked to estimate the extent of their anxiety and depression within the last week on the basis of one of four response specifications (scored 0–3). Based on the cut-off value (sum score ≥ 8) of either subscale a decision for further psycho-oncological treatment can be reached [21, 23–25].

#### QSC-R10

The short form of the questionnaire on distress in cancer patients (QSC-R10), like the long version on which it is based, is a cancer-specific self-assessment tool. Patients assess the presence and, if present, the extent of their distress using 10 items that capture cancer-specific stressors. The patients respond on a six-step Likert scale from 0 = not applicable, 1 = applies and hardly distresses me to 5 = applies and distresses me severely. The time needed for completion is approximately 3 min. The authors suggest a sum score > 14 as cut-off value for an indication for further psycho-oncological treatment [21, 26–29].

#### EORTC QLQ-C30 V3.0

QoL was assessed with a cancer-specific instrument, the Core Quality of Life Questionnaire (QLQ-C30 V3.0) of the European Organization for Research and Treatment of Cancer (EORTC). This is a self-assessment questionnaire consisting of 30 items. A global measure of QoL (Global health status; GHS) as well as five functional scales (physical, emotional, role, cognitive, social functioning) and nine symptom scales (fatigue, nausea and vomiting, pain, dyspnoea, insomnia, appetite loss, constipation, diarrhea, financial difficulties) are recorded. Patients are asked to assess their condition in terms of functional level and symptom burden during the last week on a Likert scale, ranging from 1 = not at all to 4 = very high. The global health status should also be assessed for the last week on a Likert scale ranging from 1 = very poor to 7 = excellent. For evaluation, all scales are transformed to a value range from 0 to 100, and a high value for GHS and the functional scales indicates a good QoL or a high functional level, whereas a high value for the symptom scales indicates a high symptom burden. The time for completion is approximately 11 min [30, 31]. Furthermore, there is a manual with reference values both for specific cancers and for the general population [32].

### Statistical analysis

The statistical analyses were performed with the IBM SPSS Statistics 23 package. Descriptive statistics were calculated for demographic measures, distress, and QoL. As correlation measures for metric and ordinal data, the Spearman correlation coefficient (*ρ*) was calculated. To investigate differences between groups, *t* tests for independent samples were calculated. The correspondence of classifications with more than two independent raters was calculated using Fleiss'-*κ*. Statistical tests were two-tailed with a significance level of *p* < 0.05.

## Results

### Sample characteristics

Overall, we approached 155 patients, of which a total of 109 patients could be included in the study. Of the remaining 46 patients, 40 did not return the questionnaires and six did not meet the inclusion criteria. Gender distribution was balanced with 51.4% female patients. The average age was 51.91 years (SD = 14.8). Most patients (45.9%) were diagnosed with acute myeloid leukemia (AML), 17.4% with acute lymphocytic leukemia (ALL), 14.7% with multiple myeloma (MM), 13.8% with B-cell non-Hodgkin's lymphoma (except MM), 6.4% with Hodgkin's lymphoma, and 1.8% with myelodysplastic syndrome (MDS). For further information, see Table 1.

**Table 1:** Demographic characteristics of participants

	%	*N*	*M*	SD	Min	Max
Age			51.9	14.8	18	77
Gender						
Female	51.4	56				
Male	48.6	53				
Marital status						
Married	63.3	69				
Single	14.7	16				
Permanent relationship	9.2	10				
Divorced	8.3	9				
Widowed	2.8	3				
Separated	1.8	2				
Children	69.7	76	2.1	0.8	1	4
Highest education level						
Elementary school	9.2	10				
Secondary school	9.2	10				
Abitur	6.4	7				
Vocational training	36.7	40				
University degree	32.1	35				
Other	6.4	7				
Diagnosis						
AML	45.9	50				
ALL	17.4	19				
MM	14.7	16				
B-Cell-Non-Hodgkin-Lymphoma	13.8	15				
Hodgkin-Lymphoma	6.4	7				
MDS	1.8	2				
Time since initial diagnosis (months)			15.3	35.6	0	230
Current state of disease						
Primary disease	72.5	79				
Recurrent disease	17.4	19				
Remission	8.3	9				
Secondary neoplasia	1.8	2				
Treatment						
Chemotherapy	73.4	80				
Radiotherapy	6.4	7				
Other treatment	6.4	7				
Surgery	1.8	2				
None	11.9	13				
Additional diseases	47.7	52	2.5	2.2	1	14
Previous psychological/neurological treatment	36.7	40				
Psycho-pharmaceuticals/opioids	28.4	31				
ECOG						
0	45.0	49				
1	27.5	30				
2	2.8	3				

AML = acute myeloid leukemia, ALL = acute lymphocytic leukemia, MM = multiple myeloma, MDS = myelodysplastic syndrome, ECOG = Eastern Cooperative Oncology Group performance status

### Distress

Descriptive statistics for the distress measures are presented in Table 2.

**Table 2:** Descriptive statistics for distress and quality of life

	*N*	*M*	SD	Cut-off value	*n* (%) above cut-off
PO-Bado-SF					
Fatigue/tiredness	109	1.5	1.2		
Mood swings/vulnerability/helplessness	109	1.4	1.1		
Anxiety/worries/tension	109	1.7	1.1		
Depression/grief	109	1.3	1.2		
Functional limitations in daily activities	109	1.7	1.1		
Other stressful factors (social, family related, etc.)	109	1.1	1.1		
PO-Bado-SF sum score	109	8.7	4.9	≥ 8	51 (46.8)
HADS-D anxiety	109	5.9	4.2	≥ 8	33 (30.3)
HADS-D depression	109	5.3	4.2	≥ 8	25 (22.9)
QSC-R10	109	17.1	10.3	> 14	56 (51.4)
EORTC QLQ-C30					
Global health status	109	47.6	24.2		
Physical functioning	109	61.3	29.7		
Role functioning	108	37.4	37.6		
Emotional functioning	109	62.9	25.4		
Cognitive functioning	109	67.6	30.5		
Social functioning	109	43.9	36.8		
Fatigue	109	62.2	31.7		
Nausea and vomiting	109	19.9	27.4		
Pain	109	35.6	34.4		
Dyspnoea	109	31.5	34.2		
Insomnia	109	43.4	33.8		
Appetite loss	109	47.1	41.1		
Constipation	109	29.7	35.5		
Diarrhea	109	21.7	33.8		
Financial difficulties	109	29.1	36.3		

The analysis of the externally evaluated distress with the PO-Bado-SF showed that 46.8% of the patients scored above the cut-off value. The mean distress score was 8.7 (SD = 4.9), with “restrictions of daily life” (*M* = 1.7, SD = 1.1) and “anxiety/worries/tension” (*M* = 1.7, SD = 1.1) among the highest rated items. Patients who had already undergone neurologic/psychiatric treatment (*t*_(107)_ = − 2.3, *p* = 0.024) were rated significantly more distressed than those who had not. Also, female patients were rated to be significantly more distressed than male patients (*t*_(107)_ = − 3.9, *p* < 0.001).

In the analysis of the non-cancer-specific self-assessed anxiety with the HADS-D, 30.3% of the patients scored above the cut-off. The mean score for anxiety was 5.9 (SD = 4.2). Similar values were found for depressive symptoms, here the cut-off was exceeded by 22.9% of patients, the mean score for depression was 5.3 (SD = 4.2). Single patients in HADS-D had significantly higher distress in both anxiety and depression (anxiety: *t*_(107)_ = − 2.1, *p* = 0.038; depression: *t*_(107)_ = 2.2, *p* = 0.033) than patients who lived in a relationship.

The evaluation of distress with the cancer-specific QSC-R10 showed in 51.4% of the patients a value above the cut-off. Here the mean score was 17.1 (*SD* = 10.3). As with the PO-Bado-SF, in the QSC-R10 patients who had a history of neurologic/psychiatric treatment were significantly more distressed (*t*_(107)_ = − 2.9, *p* = 0.004) than those who had not.

As expected, the different screening measures correlated highly (*ρ* > 0.50) with each other (Table 3). The self-assessment methods (HADS-D and QSC-R10) correlated most strongly with each other (*ρ* > 0.70). Although, the cancer-specific PO-Bado-SF correlated more strongly with the cancer-specific QSC-R10 (*ρ* = 0.569, *p* > 0.001) than with the non-cancer-specific HADS-D (anxiety: *ρ* = 0.556, *p* > 0.001; depression: *ρ* = 0.538, *p* > 0.001), those differences in effect sizes were not significant (0.569 vs. 0.556 *p* = 0.889, 0.569 vs. 0.538 *p* = 0.744).

**Table 3:** (Inter-) correlations Spearman’s Rho (*ρ*): distress and QoL

	PO-Bado-SF	HADS-D Anxiety	HADS-D Depression	QSC-R10
HADS-D anxiety	.556**			
HADS-D depression	.538**	.751**		
QSC-R10	.569**	.716**	.773**	
Global health status	− .324**	− .325**	− .472**	− .472**
Physical functioning	− .315**	− .311**	− .502**	− .548**
Role functioning	− .255**	− .214*	− .374**	− .310**
Emotional functioning	− .568**	− .646**	− .658**	− .637**
Cognitive functioning	− .322**	− .394**	− .511**	− .5.32**
Social functioning	− .221*	− .311**	− .528**	− .462**
Fatigue	.287**	.229*	.435**	.413**
Nausea and vomiting	.293**	.200*	.393**	.318**
Pain	.226*	.185^n.s^	.351**	.348**
Dyspnoea	.193*	.232*	.313**	.345**
Insomnia	.378**	.406**	.431**	.388**
Appetite loss	.203*	.177^n.s^	.320**	.247**
Constipation	.160^n.s^	.328**	.258**	.231*
Diarrhea	.312**	.184^n.s^	.333**	.334**
Financial difficulties	.157^n.s^	.338**	.286**	.316**

*p < .05; **p < .01

**Fig. 1:**
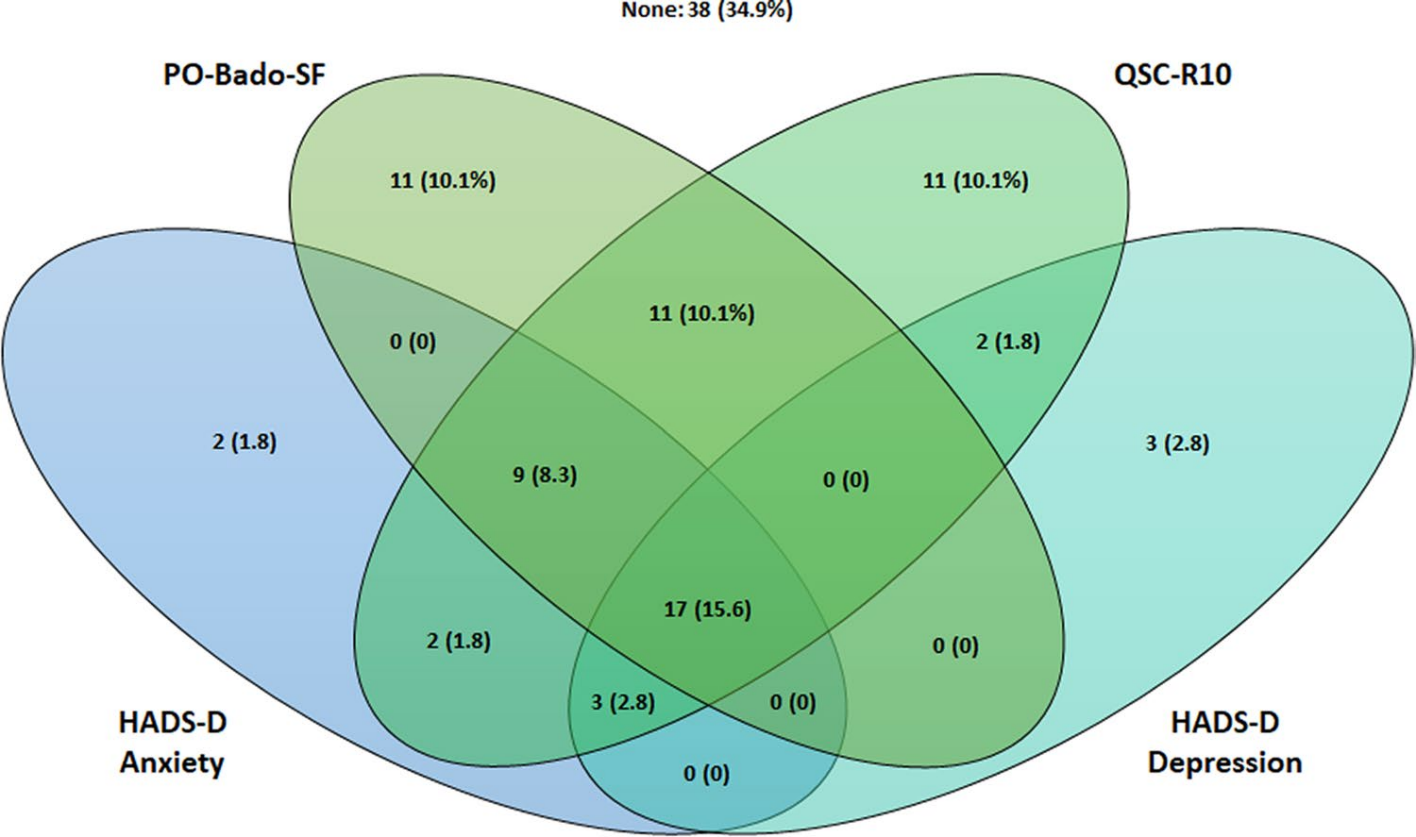
Venn diagram of patient classification as highly distressed (i.e., scoring above the cut-off by screening instrument and combinations thereof)

Figure 1 gives an overview of how the different screening instruments or their combination classified patients as highly distressed (i.e., scoring above the respective cut-off). Overall, 34.9% of patients did not score above the cut-off of either screening instrument and 15.6% were classified by all instruments as highly distressed. The PO-Bado-SF and the QSC-R10 were the only screening instruments in which a substantial number of patients (10.1% in each) scored above the cut-off without scoring above the cut-off in any of the other screening instruments. Additionally, the combination of PO-Bado-SF and QSC-R10 yielded another 10.1% of patients that did not exceed the cut-off value in any of the HADS-D scales (Fig. 1). In total, the agreement of classification by the four screening measures was moderately strong (Fleiss*'-κ* = 0.4, *p* < 0.001).

Patients classified by either one or a combination of the screening instruments as highly distressed (i.e., scoring above the cut-off) differed significantly from less/non-distressed patients (i.e., scoring below the cut-off). Effect sizes (Cohen’s *d*) ranged from 0.83 to 1.35 indicating strong effects. Among the distress measures only the item “Other stressful factors (social, family related, etc.)” of the PO-Bado-SF did not reach significance in the group comparisons (Table 4).

**Table 4:** Descriptive statistics, *t*-Test results, and effect sizes (Cohen’s *d*) comparing highly and less/non-distressed patients (i.e., scoring above or below the cut-off of any screening instrument)

	Patients scoring above cut-off			Patients scoring below cut-off						
	*N*	*M*	SD	*N*	*M*	SD	*df*	*t*	*p*	Cohen’s *d*
PO-Bado-SF										
Fatigue/tiredness	71	1.8	1.2	38	0.8	0.8	98.51	− 5.232	> .001	0.83
Mood swings/vulnerability/helplessness	71	1.8	1.1	38	0.6	0.7	102.69	− 7.483	> .001	1.09
Anxiety/worries/tension	71	2.1	0.9	38	1.0	0.9	107	− 5.553	> .001	1.00
Depression/grief	71	1.7	1.1	38	0.6	0.8	102.30	− 6.159	> .001	0.92
Functional limitations in daily activities	71	2.1	1.0	38	1.0	0.8	107	− 5.803	> .001	1.00
Other stressful factors (social, family related, etc.)	71	1.2	1.1	38	0.8	0.9	107	− 1.873	.064	0.36
PO-Bado-SF sum score	71	10.7	4.5	38	4.8	2.6	105.98	− .8.716	> .001	1.20
HADS-D anxiety	71	7.6	3.9	38	2.7	2.1	106.99	− 8.353	> .001	1.16
HADS-D depression	71	7.0	4.2	38	2.2	1.8	102.81	− 8.440	> .001	1.14
QSC-R10	71	21.9	9.3	38	8.0	3.6	99.84	− 11.121	> .001	1.35
EORTC QLQ-C30										
Global health status	71	41.8	21.6	38	58.6	25.2	107	3.639	> .001	0.69
Physical functioning	71	54.9	30.2	38	73.2	24.9	88.9	3.364	.001	0.62
Role functioning	70	32.4	37.6	38	46.5	36.2	106	1.886	> .001	0.38
Emotional functioning	71	54.7	25.3	38	78.5	17.2	100.82	5.817	> .001	0.94
Cognitive functioning	71	61.9	31.3	38	78.1	26.3	107	2.701	.008	0.53
Social functioning	71	36.9	37.3	38	57.0	32.3	107	2.815	.006	0.55
Fatigue	71	67.9	30.1	38	51.5	31.9	107	− 2.659	.009	0.52
Nausea and vomiting	71	23.9	27.4	38	12.3	25.9	107	− .2.157	.033	0.42
Pain	71	39.7	35.0	38	28.1	32.4	107	− 1.691	.094	0.34
Dyspnoea	71	38.5	35.0	38	18.4	28.7	107	− 3.029	.003	0.59
Insomnia	71	49.3	34.7	38	32.5	29.5	107	− 2.541	.012	0.49
Appetite loss	71	51.2	41.7	38	39.5	39.4	107	− 1.422	.158	0.28
Constipation	71	32.9	36.7	38	23.7	32.8	107	− 1.289	.200	0.26
Diarrhea	71	27.7	37.4	38	10.5	22.1	105.89	− 3.013	.003	0.51
Financial difficulties	71	34.3	38.6	38	19.3	29.6	107	− 2.083	.040	0.41

### Quality of life

Descriptive statistics for the QoL measures are presented in Table 2.

The self-assessed QoL (GHS) was on average 47.6 (SD = 24.2). Physical, emotional, and cognitive functioning were rated better than the GHS, but role and social functioning were rated worse than the GHS. Fatigue, followed by appetite loss, and insomnia were the symptoms most often reported by patients (Table 2).

Both the GHS and the functional scales all correlate significantly negatively with the measures of distress. As expected, the symptom scales correlate positively with the distress measures (Table 3).

#### Comparison of highly and less/non-distressed patients regarding QoL

Regarding their QoL highly distressed patients differed significantly from less/non-distressed patients in their GHS and all functional scales as well as the symptom scales except pain, appetite loss, and constipation (Table 5). The effect size for the difference in GHS was 0.69, which indicates a medium effect. For the functional scales the effect sizes ranged from medium effects for role (0.38), cognitive (0.53), social (0.55), and physical functioning (0.62) to 0.94 which indicates a strong effect for emotional functioning. In the symptom scales the effect sizes ranged from 0.41 for financial difficulties, 0.42 for nausea and vomiting, 0.49 for insomnia, 0.51 for diarrhea, and 0.52 for fatigue, to 0.59 for dyspnea, all indicating medium effects (Table 4).

## Discussion

Patients affected by hematologic malignancies often have elevated distress levels compared to patients with other cancers [2, 6, 8–11]. At the same time, QoL is reduced, functioning is restricted and symptom burden increased [15]. If the psychosocial stress factors are not or insufficiently assessed, this can lead to unrecognized support needs or in the worst case even a negative effect on patients’ mortality [7, 33–35]. It is therefore of particular importance to be able to provide a timely indication for further psycho-oncological treatment, especially in such a highly distressed patient population. Therefore, the aim of this study was to determine the extent of distress in patients with hematologic malignancies and their QoL. In addition, the relationship between distress and QoL was investigated. In particular, we looked at to what extend patients classified as highly distressed (scoring above the cut-off of at least one screening instrument) differed from less/non-distressed patients (scoring below the cut-off of all screening instruments).

### Clinical implications

#### Psychosocial distress

The present study shows that a sizeable portion of patients with hematologic malignancies is highly distressed. Depending on the screening instrument used the fraction of patients that scores above the respective cut-off value varies from slightly under one-fourth to over half of patients. This corroborates the current state of relevant studies [2, 6, 8–11]. Nevertheless, there were clear differences between the instruments with regard to the number of patients whose distress scores exceeded the cut-off values of the respective screening instruments. The non-cancer-specific instrument (HADS-D) classified, depending on the criterion, 30.3% (anxiety) or 20.9% (depression) of the patients as so severely affected, that further psycho-oncological treatment would be indicated. For cancer-specific instruments, the proportion of patients classified this way was markedly higher: 46.8% for PO-Bado-SF and even 51.4% for QSC-R10. It is reasonable to assume that an inquiry aimed at cancer-specific stress factors leads to a more precise evaluation compared with more general psychic stressors and thus to more recognition of distress in this population [26]. The HADS was constructed with somatically ill patients in mind and therefore excluding vegetative symptoms of depression, like appetite loss or physical weakness, not to overestimate the prevalence of depressive symptoms in this population. The exclusion of nearly all somatic symptoms restricts the HADS to the symptom complexes of anxiety and depression, this limits somewhat a holistic assessment of distress as defined by the NCCN guidelines. Cancer-specific instruments, like the QSC or the PO-Bado, were explicitly constructed with a focus on experientially oriented item formulations that are relevant to daily life experiences of cancer patients also including typical somatic symptoms. The aim of this was to improve clinical relevancy and also being able to derive indications for treatment [21]. Additionally, the PO-Bado as an external assessment tool provides the possibility to incorporate for example non-verbal aspects of the patient’s expression of distress and thereby utilizes sources of information not accessible to self-rating instruments [20, 36]. Senf et al., who also used the PO-Bado-SF, found that 56.3% of the patients scored above the cut-off value, albeit all tumor entities were included and the proportion of female patients was higher [37]. Furthermore, our study found some demographic variables that significantly differentiated patients with respect to their distress. However, this was partly dependent on the instruments used. The relationship status was reliable only for HADS-D as an indicator, whereas a history of neurological/psychiatric treatment only played a role for cancer-specific instruments (PO-Bado-SF, QSC-R10). This replicates some results of Senf et al., where previous psychiatric treatment was accompanied by greater distress [37]. Gender made a difference only with the PO-Bado-SF, with women being more distressed. This might be due to a statistical artifact or a bias in the raters. There are similar findings reported in the literature [38–40], but not only pertaining to the PO-Bado. It seems that generally women are more distressed than men. This phenomenon needs further explanation for up to now there only exist common sense explanations (response bias, biological, social and demographic influences, differences in coping styles and fear processing) none of which has been scientifically studied in relation to cancer [41]. The correlations between the individual screening instruments were consistently strong (*ρ* > 0.50), which indicates that the same underlying construct (distress) is assessed with all instruments. The fact that the self-evaluation instruments correlate more strongly with each other (*ρ* > 0.70) is probably due to the common method variance. Nevertheless, it can also be seen that the PO-Bado-SF as the only external assessment instrument correlates most strongly with the also cancer-specific QSC-R10, but this difference is only slight and does not reach significance. Nevertheless, if replicated in a bigger sample, it could be assumed that such a difference is due to domain specificity as underlying factor, meaning that the cancer-specific instruments concurringly tap into the same central problem areas. It is also remarkable, that there was a substantial number of patients that exceeded the cut-off and thus had an indication for further psycho-oncological treatment only in the cancer specific screening instruments. Taken together this number amounts to nearly a third of patients. This seems to imply that domain specificity is a factor not to be neglected when choosing screening instruments. Furthermore, the fact that highly distressed patients differed markedly in their psycho social burden, reflected mainly in symptoms of anxiety and depression, from those who did not exceed the cut-off values, suggests the usefulness of these cut-offs in clinical practice.

#### Quality of life

The QoL of patients with hematologic malignancies is significantly reduced in comparison to patients with other cancers and, of course, the general population. This applies not only to the GHS, but essentially to all functional and symptom scales, as a comparison with relevant reference populations and the general population published by Scott et al. confirms [32]. The study population clearly falls short of the cut-off values established by Giesinger et al. for clinical relevance of functional restrictions (i.e., physical = 83 and emotional = 70 vs. 61 and 63 in the study population) and likewise exceeds those cut-offs for symptom burdens (pain = 25 and fatigue = 39 vs. 36 and 62 in the study population) [42]. In this respect, the generally observed medium-to-strong correlations between the measures of distress and those of QoL are not surprising, but support the notion of a close relation between those two factors. With regard to the problem scales, patients suffer most from fatigue, appetite loss, and insomnia. Those conditions often occur in the course of chemotherapy, the form of therapy with which most patients with hematologic malignancies are treated. In addition, there seems to be a connection between this symptom complex and distress, as our data and data from other studies show [43]. Likewise, role and social functioning are particularly affected by the often protracted inpatient stays, sometimes in isolation, which drag patients out of their everyday life and their social environment. Finally, the present study showed that patients scoring above the cut-off value of either of the screening instruments (highly distressed patients) differed significantly from less/non-distressed patients with regard to their QoL. This complements the existent literature on the close relation between distress and QoL [15, 16, 44]. The effect sizes for those differences are mostly medium but with a strong effect for emotional functioning, which suggests a clinical relevance in general and points to the necessity of focusing on emotional factors.

## Limitations

One limitation of the study is that the study population comes from a convenience sample. This is associated with a typical set of disadvantages. Foremost, the lack of generalizability of the study results should be mentioned here. Nevertheless, the demographic data of our study population are comparable with other populations that also originate from the acute setting [16, 17]. The heterogeneity of hematologic malignancies, treatment forms, and stages within the study population reflects the naturalistic study design and the actual conditions prevailing in a hematologic-oncological ward of an acute hospital. Due to the large number of statistical tests in the explorative approach of the study, there is an increased probability of alpha error accumulation. Of course, the measures used in this study also have limitations. For the self-report measures (HADS-D, QSC-R10) these are the commonly encountered biases like social desirability, exaggeration or attenuation effects. Nevertheless, all chosen measures have proved their reliability and validity in numerous studies. Another problem might be the occurrence of memory effects, which we tried to avoid by scheduling fixed appointments for the return of the questionnaires at most three days after the initial interview. The reliability and validity of the external assessment (PO-Bado-SF) relied on the experience of the interviewer. Therefore, all interviewers received intensive training in conducting the interviews and rating patients and trial interviews were conducted to reach consistent ratings. Self-selection is a problem that cannot be avoided completely in studies based on a convenience sample. The exclusion of patients without sufficient command of the German language is a further restricting factor, but only three patients were excluded because of lack of language capabilities. Unfortunately, we cannot give a precise account of the number of patients with a comorbid psychiatric disorder, but are only able to state the number of patients with previous psychological/ neurological treatment.

## Conclusion

Screening instruments represent a valuable diagnostic tool for providing an indication for further psycho-oncological treatment. In comparison, however, the cancer-specific instruments seem to be able to identify patients that need further psycho-oncological treatment (e.g., further diagnostics, psycho education, counseling) even more specifically because of their targeted orientation towards the needs and requirements of this particular population. This should be considered when selecting appropriate measures, especially under the premise that patients identified as requiring treatment should then also be guaranteed subsequent psychosocial care [19]. In the present study the cancer-specific instruments not only covered the psychosocial stress aspects more specifically, but also allowed a more differentiated presentation of the stress aspects in the area of QoL. Overall, this is a clear indication of the close link between distress and the more far-reaching aspects of QoL. It therefore seems sensible to keep this in mind during further treatment in order to be able to pursue a holistic approach in the treatment of these patients and thus optimize the quality of treatment.

## Data Availability

The data that support the findings of this study are available on request from the corresponding author. The data are not publicly available due to privacy or ethical restrictions.
